# Prognostic significance of uncertain resection for metastasis in the highest mediastinal lymph node after surgery for clinical N0 non-small cell lung cancer

**DOI:** 10.3389/fsurg.2023.1115696

**Published:** 2023-06-15

**Authors:** Valentina Marziali, Luca Frasca, Vincenzo Ambrogi, Alexandro Patirelis, Filippo Longo, Pierfilippo Crucitti

**Affiliations:** ^1^ Department of Thoracic Surgery, University Tor Vergata, Rome, Italy; ^2^ Department of Thoracic Surgery, University Campus Bio-Medico, Rome, Italy; ^3^ Microbiology, Immunology, Infectious Diseases, and Transplants (MIMIT), University Tor Vergata, Rome, Italy

**Keywords:** non-small cell lung cancer (NSCLC), R classification, complete resection, uncertain resection, high mediastinal lymph node metastases, pN2 disease

## Abstract

**Background:**

The International Association for the Study of Lung Cancer defined types of surgical resection and considered the positivity of the highest mediastinal lymph node resected a parameter of “uncertain resection” (R-u). We investigated the metastases in the highest mediastinal lymph node, defined as the lowest numerically numbered station among those resected. We aimed to evaluate the prognostic value of R-u compared with R0.

**Materials and methods:**

We selected 550 patients with non-small cell lung cancer at clinical Stage I, IIA, IIB (T3N0M0), or IIIA (T4N0M0) undergoing lobectomy and systematic lymphadenectomy between 2015 and 2020. The R-u group included patients with positive highest mediastinal resected lymph node.

**Results:**

In the groups of patients with mediastinal lymph node metastasis, we defined 31 as R-u (45.6%, 31/68). The incidence of metastases in the highest lymph node was related to the pN2 subgroups (*p* < 0.001) and the type of lymphadenectomy performed (*p* < 0.001). The survival analysis compared R0 and R-u: 3-year disease-free survival was 69.0% and 20.0%, respectively, and 3-year overall survival was 78.0% and 40.0%, respectively. The recurrence rate was 29.7% in R0 and 71.0% in R-u (*p*-value < 0.001), and the mortality rate was 18.9% and 51.6%, respectively (*p*-value < 0.001). R-u variable showed a tendency to be a significant prognostic factor for disease-free survival and overall survival (hazard ratio: 4.6 and 4.5, respectively, *p*-value < 0.001).

**Conclusions:**

The presence of metastasis in the highest mediastinal lymph node removed seems to be an independent prognostic factor for mortality and recurrence. The finding of these metastases represents the margin of cancer dissemination at the time of surgery, so it could imply metastasis into the N3 node or distant metastasis.

## Introduction

According to the European Society for Medical Oncology (ESMO), the treatment for clinical Stage I, IIA, IIB (T3N0M0), or IIIA (T4N0M0) non-small cell lung cancer (NSCLC) is the anatomic surgical resection of the involved lobe together with systematic node dissection (1, 2). The resection status after surgery has been proven to be an important predictor of prognosis and has an impact on the choice of further treatments. The residual tumor (R) classification includes: R0 (no residual tumor), R1 (microscopic residual tumor), and R2 (macroscopic residual tumor) (3). The International Association for the Study of Lung Cancer redefined the resection status into the categories: complete resection, incomplete resection, and uncertain resection. The last one included all cases without microscopic disease on resection margins but with one of the following criteria: lymphadenectomy less rigorous than systematic or lobe-specific nodal dissection, positive highest mediastinal node removed, carcinoma *in situ* on the bronchial margin, or positive pleural lavage cytology (4). Few previous studies focused attention on the impact of metastases in the highest mediastinal lymph node (HMLN) removed on prognosis. Moreover, the definitions of HMLN varied among these studies, leading to differences in patient selection and survival analysis. IASLC considered the highest mediastinal lymph node among those resected (3, 5).

We retrospectively reviewed patients who underwent lobectomy and systematic lymph node dissection. In patients with mediastinal nodal metastases, we investigated those who were HMLN positive. To avoid bias of the differences in the dissection of the right and left mediastinal nodal stations, because of anatomical difference (6), and according to IASLC, we defined HMLN as the lowest numerically numbered station among resected lymph node stations. These patients were defined as R-uncertain (R-u). We aimed to evaluate the prognostic value of R-u, compared with R0, in a population of patients with mediastinal node metastases.

## Material and methods

### Patient selection

We retrospectively reviewed 550 patients with NSCLC Stage I, IIA, IIB (T3N0M0), or IIIA (T4N0M0) who underwent lobectomy with systematic lymphadenectomy between January 2015 and December 2020. We excluded synchronous cancer or history of another cancer; neoadjuvant chemotherapy and/or radiotherapy; neuroendocrine lung tumors benign neoformations; cN2 or cM1-M1; R1–2 resection; segmentectomy, wedge, and pneumonectomy; and lymph node sampling. Other causes of “uncertain resection” as carcinoma *in situ* on the bronchial margin or positive pleural lavage cytology were also excluded.

After surgery, the follow-up consisted of a computed tomography (CT) scan at 6 months for the first 2 years and then at 12 months. The median time for follow-up was 26 months (range 12–72 months).

Preoperative staging was achieved by CT scan and synchronized CT with 18-fluorodeoxyglucose-positron emission tomography (18FDG-PET/CT) scanning, dated no more than 30 days. Before surgery, the histologic diagnosis was obtained by CT-guided transthoracic biopsy or intraoperative frozen section. Endobronchial ultrasound (EBUS) biopsy was performed for the suspected lymph node: diameter greater than 10 mm in the short axis at CT scan (7) or standardized uptake value (SUV) max score greater than 2.0 at 18FDG-PET/CT (8). Negative histologic biopsies on suspected lymph nodes were considered cN0. Invasive lymph node staging was executed if the tissue from the endobronchial biopsy was inadequate for the histological diagnosis. The choice between mediastinoscopy and thoracoscopy was guided by lymph node position.

All patients underwent lobectomy with hilar and mediastinal lymphadenectomy through thoracotomy (posterior or anterolateral) or video-assisted thoracoscopic surgery (VATS).

Pleural lavage cytology was performed in all patients to detect those with “uncertain resection” and could focalize the attention on R-u for metastases in the highest mediastinal lymph node. Systematic nodal dissection was carried out in all patients sampling at least three mediastinal lymph node stations (always including station 7) (9). If the lymphadenectomy did not fulfill the criteria of systematic nodal dissection was considered sampling.

Whenever possible, lymph nodes were resected *en bloc* with the surrounding fat. If a lymph node was fragmented, all parts were considered as the same node station for the histological analysis. The number of resected lymph nodes was evaluated in every patient as the sum of lymph nodes located within the resected lobe and the others resected during lymphadenectomy. If lymph nodes were fragmented, each fragment was counted as another lymph node.

The pathological classification was based on the 2015 World Health Organization Classification of Lung Cancer and pathological staging was based on the 8th edition of the lung cancer TNM (Tumor Node Metastases) (10, 11).

### R classification

According to the new category of resection proposed by IASLC (4), the cohort of patients was reassigned to the R-u category if they met at least one of the following criteria: lymphadenectomy less rigorous than systematic, metastases on the highest mediastinal lymph node resected, pleural lavage cytology positive, or carcinoma *in situ* in the bronchial margin. We included only systematic lymph node dissection in our population to avoid selection bias.

To focus attention on the role of metastasis in the highest mediastinal lymph node, patients with positive pleural lavage cytology or carcinoma *in situ* on the bronchial margin were excluded from the R-u group. Finally, the R-u category was composed of only patients with positive higher mediastinal nodes.

### Statistical analysis

Statistical data analysis was conducted using the SPSS Statistics program version 26.0 (IBM Corp., Armonk, NY, United States). Student’s T test was used for continuous variables and Pearson’s chi-squared test for discontinuous variables. The threshold of significance was set at *p*-value = 0.050. The major outcomes for survival were overall survival (OS) and disease-free survival (DFS)). OS was calculated as the time from surgery to death or last follow-up. DFS was defined from surgery to the evidence of relapse or metastasis. Survival was graphically represented with Kaplan–Meier curves. Independent prognostic factors for OS and DFS were then evaluated with a Cox proportional hazard regression model. Univariate analysis and multivariate analysis, using the backward stepwise method, were carried out with the variables that influenced the various survivals.

## Results

We selected 550 patients with clinical N0 lung cancer. The baseline characteristics are presented in Table 1*.* The mean age was 69.70 years (SD 8.2), 343 patients (62.4%) were male and 454 (82.5%) were current or past smokers. The patient’s distribution for pT and pN classifications were as follows: pT1 296 (53.8%), pT2 174 (31.6%), pT3 58 (10.6%), and pT4 22 (4.0%); pN0 426 (77.5%), pN1 56 (10.2%), and pN2 68 (12.3%). The pN2 classification was divided into subgroups: pN2a2 39 (57.4%); pN2a1, or skip metastases, 15 (22.1%); and pN2b 14 (20.6%). The most frequent histology was adenocarcinoma (442 patients, 80.4%). VATS was performed in 385 patients (70.0%). The mean number of resected lymph nodes was 19.50 (SD 13.1), while the mean number of lymph node ratio was 22.60 (SD 17.0). The right upper lobe was the most frequently affected lobe (36.4%) while the right middle lobe was the less affected one (3.6%). Regarding the R classification in the whole population, 31 patients (5.6%) were R-u while the remaining 519 (94.4%) were R0.

**Table 1:** Clinical and pathological characteristics of the cohort population.

Variables	Total
Age	69.7 (SD 8.2)
Sex	
Male	343 (62.4%)
Female	207 (34.3%)
Smoke habit	
Non-smoker	95 (17.3%)
Smoker	454 (82.5%)
pT	
1	296 (53.8%)
2	174 (31.6%)
3	58 (10.6%)
4	22 (4.0%)
pN	
0	426 (77.5%)
1	56 (10.2%)
2	68 (12.3%)
pN2 subgroups	
2 a2	39 (57.4%)
2 a1	15 (22.1%)
2 b	14 (20.6%)
Histology	
Adenocarcinoma	442 (80.4%)
Squamous cell carcinoma	108 (19.6%)
Open	385 (70.0%)
VATS	165 (30.0%)
No. lymph nodes	19.5 (SD 13.1)
Lymph node ratio	22.6 (SD 17.0)
Lobe	
RUL	200 (36.4%)
RML	20 (3.6%)
RLL	106 (19.3%)
LUL	136 (24.7%)
LLL	88 (16.0%)
Tumor diameter at CT	27.9 (SD 17.0)
SUVmax tumor	
< 5	265 (48.2%)
> 5	285 (51.8%)
Lymph node diameter at CT	
<1 cm	494 (89.8%)
>1 cm	56 (10.2%)
SUVmax mediastinal lymph nodes	
<2	506 (92.0%)
>2	44 (8.0%)
R classification	
R-u	31 (5.6%)
R0	519 (94.4%)

CT, computed tomography; LLL, left lower lobe; LUL, left upper lobe; RML, right middle lobe; RLL, right lower lobe; RUL, right upper lobe; VATS, video-assisted thoracic surgery; SD, standard deviation; SUV, standardize.

The association between R classification and clinical variables in the pN2 group is displayed in Table 2. The lymph node ratio (LNR) was evaluated as the ratio between positive lymph nodes and all resected lymph nodes. The incidence of metastases in the highest mediastinal lymph node was related to the pN2 subgroups (*p* < 0.001). No relation was found for pT classification (*p*-value = 0.60), histology (*p*-value = 0.94), number of resected lymph nodes (*p*-value = 0.31), lymph node ratio (*p*-value = 0.18), affected lobe (*p*-value = 0.42), and tumor diameter (*p*-value = 0.62).

**Table 2:** Association between R classification and clinical variables in the pN2 group.

Variables	Total	R0 (*n*, 37)	R-u (*n*, 31)	*p*-value
Age	69.7 (SD 8.2)	68.1 (SD 7.9)	73.0 (SD 6.7)	0.71
Sex				0.52
Male	41 (60.3%)	21 (56.8%)	20 (64.5%)	
Female	27 (39.7%)	16 (43.2%)	11 (35.5%)	
Smoke habits				0.52
Non-smoker	11 (16.2%)	5 (13.5%)	6 (19.4%)	
Smoker	57 (83.8%)	52 (86.5%)	25 (80.6%)	
pT				0.6
1	26 (38.2%)	13 (35.1%)	13 (41.9%)	
2	34 (50%)	20 (54.1%)	14 (45.2%)	
3	7 (10.3%)	4 (10.8%)	3 (9.7%)	
4	1 (1.5%)	0 (0.0%)	1 (3.2%)	
pN subgroups				**<0**.**001**
2a2	39 (57.4%)	30 (81.1%)	9 (29.0%)	** **
2a1	15 (22.0%)	2 (5.4%)	13 (42.0%)	** **
2b	14 (20.6%)	5 (13.5%)	9 (29.0%)	** **
Histology				0.94
Adenocarcinoma	59 (86.8%)	32 (86.5%)	27 (87.1%)	
Squamous cell carcinoma	9 (13.2%)	5 (13.5%)	4 (12.9%)	
Open	16 (23.5%)	6 (16.2%)	10 (32.3%)	0.12
VATS	52 (76.5%)	31 (83.8%)	21 (67.7%)	
No. lymph nodes	19.5 (SD 13.1)	21.5 (SD 12.3)	19.1 (SD 10.6)	0.31
Lymph node ratio	22.6 (SD 17.0)	21.5 (SD 12.4)	24.1 (SD 15.3)	0.18
Lobe				0.42
RUL	24 (35.3%)	11 (29.7%)	13 (41.9%)	
RML	4 (5.9%)	2 (5.4%)	2 (6.5%)	
RLL	17 (25.0%)	9 (24.3%)	8 (25.8%)	
LUL	16 (23.5%)	12 (32.4%)	4 (12.9%)	
LLL	7 (10.3%)	3 (8.1%)	4 (12.9%)	
Tumor diameter at CT	27.9 (SD 17.0)	26.4 (SD 9.3)	27.2 (SD 12.6)	0.62
SUVmax tumor				0.12
< 5	5 (7.4%)	1 (2.7%)	4 (12.9%)	
> 5	63 (92.6%)	36 (97.3%)	27 (87.1%)	
Lymph node diameter at CT				0.12
<1 cm	52 (76.5%)	31 (83.8%)	21 (67.7%)	
>1 cm	16 (23.5%)	6 (16.2%)	10 (32.3%)	
SUVmax mediastinal lymph nodes				0.31
<2	48 (70.6%)	28 (75.7%)	20 (64.5%)	
>2	20 (29.4%)	9 (24.3%)	11 (35.5%)	

CT, computed tomography; LLL, left lower lobe; LUL, left upper lobe; RML, right middle lobe; RLL, right lower lobe; RUL, right upper lobe; VATS, video-assisted thoracic surgery; SD, standard deviation; SUV, standardize uptake value.

Bold indicate statistical significative value.

Table 3 shows the topographic distribution of lymph node metastasis for each affected lobe. In the right upper lobe, station 2 was the most frequent highest positive lymph node station (53.8%), while station 4 was the most frequent (100%) for the right middle lobe. In the right lower lobe, the highest positive station was found in station 7 (50.0%). Station 4 was the most common (50.0%) for the left upper lobe. In the left lower lobe, station 5 and station 7 were equally common (50.0%).

**Table 3:** Topographic distribution of lymph node metastasis for each affected lobe.

Positive stations	RUL	RML	RLL	LUL	LLL
2	7 (53.8%)	0 (0.0%)	1 (12.5%)	0 (0.0%)	0 (0.0%)
4	6 (46.2%)	2 (100%)	3 (37.5%)	2 (50%)	0 (0.0%)
5	0 (0.0%)	0 (0.0%)	0 (0.0%)	1 (25.0%)	2 (50.0%)
6	0 (0.0%)	0 (0.0%)	0 (0.0%)	1 (25.0%)	0 (0.0%)
7	0 (0.0%)	0 (0.0%)	4 (50.0%)	0 (0.0%)	2 (50.0%)
8	0 (0.0%)	0 (0.0%)	0 (0.0%)	0 (0.0%)	0 (0.0%)
9	0 (0.0%)	0 (0.0%)	0 (0.0%)	0 (0.0%)	0 (0.0%)
Total	**13**	**2**	**8**	**4**	**4**

LLL, left lower lobe; LUL, left upper lobe; RML, right middle lobe; RLL, right lower lobe; RUL, right upper lobe.

Bold indicate statistical significative value.

**Figure 1:**
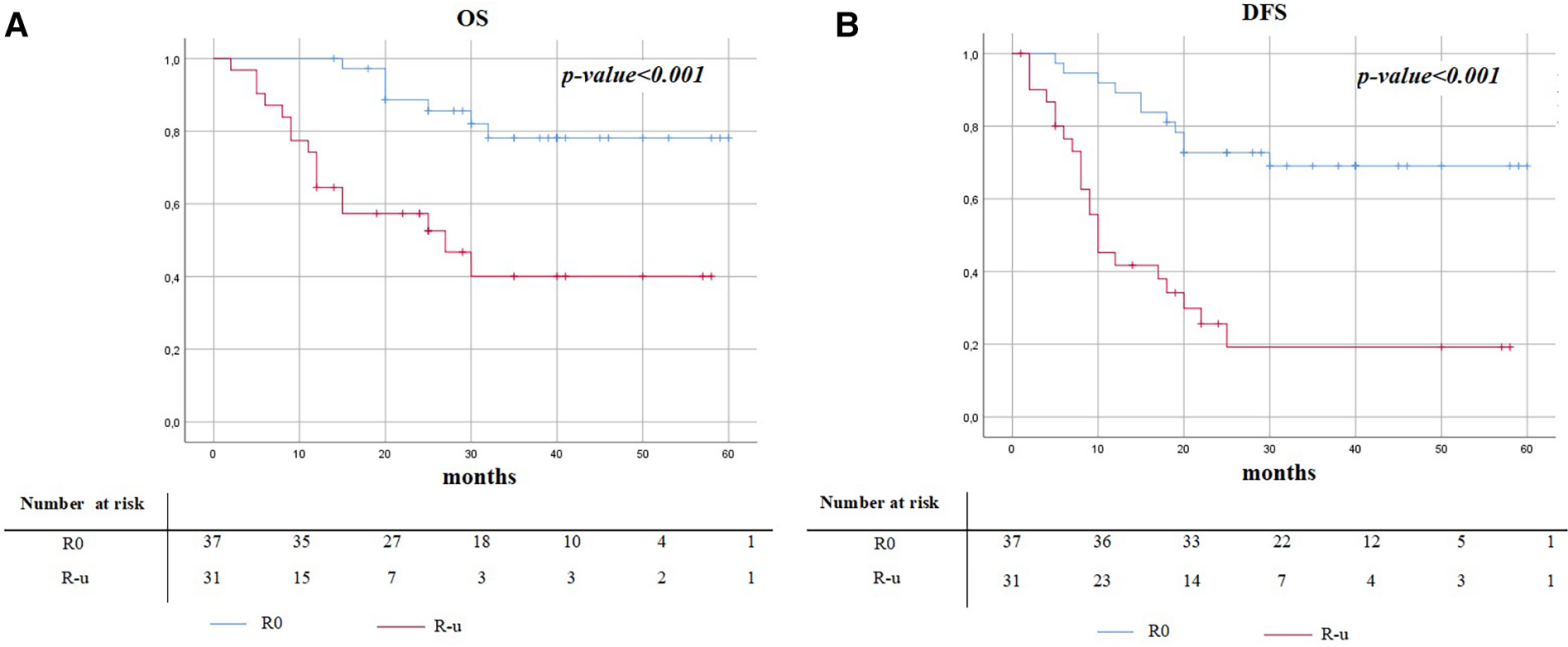
Kaplan–Meier survival curves of overall survival (**A**) and disease-free survival (**B**) between R0 and R-u.

The median follow-up time was 33.9 months (SD 14.8). Postoperative survival analysis in patients with N2 disease, comparing R0 and R-u resections, is shown in Table 4. Kaplan–Meier curves are illustrated in Figure 1. The recurrence rate was 29.7% (11/37) in the R0 group, while in the R-u group it was 71.0% (22/31); 3-year DFS was 69.0% (mean time to relapse of 15.5 ± 7) and 20.0% (mean time to relapse of 9.9 ± 6.6), respectively. The mortality rate was 18.9% (7/37) in the R0 group, while in the R-u group it was 51.6% (16/31); 3-years OS was 78.0% (mean time to relapse of 23.1 ± 6.) and 40.0% (mean time to relapse of 12.7 ± 8.2), respectively.

**Table 4:** Survival analysis in patients with N2 disease comparing R0 and R-u resections.

Group	*N*	Recurrence	3-year DFS	Mean time to relapse (Months ± SD)	Mortality	3-year-OS	Mean time to death (months ± SD)
R0	37	11 (29.7%)	69.0%	15.5 ± 7.1	7 (18.9%)	78.0%	23.1 ± 6.1
R-u	31	22 (71.0%)	20.0%	9.9 ± 6.6	16 (51.6%)	40.0%	12.7 ± 8.2

DFS, disease-free survival; OS, overall survival; SD, standard deviation.

The univariate analysis for DFS and OS, in the N2 population, included the variable listed in Table 5. The R-u variable showed a tendency to be a significant prognostic factor. The hazard ratio (HR) for DFS and OS of the R-u group was higher if compared with the R0 group [DFS: HR 4.6 (95% CI 2.2–9.6), *p*-value < 0.001; OS: HR 4.5 (95% CI 1.8–10.9), *p*-value < 0.001]. The multivariable analysis was evaluated using variables with a significant *p*-value at univariate analyses (Table 6). R-us remained a significant prognostic factor for DFS and OS [DFS: HR 3.2 (95% CI 1.4–7.4), *p*-value = 0.008; OS: HR 2.0 (95% CI 0.7–6.1), *p*-value < 0.001].

**Table 5:** The univariate analysis for DFS and OS in the N2 population.

Univariate analysis	DFS		OS	
	HR (95% CI)	*p*-value	HR (95% CI)	*p*-value
Age	1.1 (1.0–1.1)	**0**.**04**	1.0 (1.0–1.5)	**0**.**02**
Sex	0.9 (0.5–1.9)	0.83	0.6 (0.2–1.4)	0.24
Smoke	0.4 (0.2–0.9)	0.06	0.9 (0.3–2.9)	0.96
pT	1.8 (1.2–2.9)	**0**.**02**	2.3 (1.3–4.0)	**0**.**005**
pN2 subgroups	1.9 (1.3–2.8)	**0**.**001**	1.9 (1.2–3.0)	**0**.**006**
Histology	1.5 (0.6–3.6)	0.39	1.9 (0.7–5.1)	0.20
Open vs. VATS	1.9 (0.9–3.9)	0.09	1.5 (0.6–3.5)	0.41
No. lymph nodes	0.9 (0.9–1.0)	0.12	0.9 (0.9–1.0)	0.75
Lymph node ratio	1.0 (1.0–1.0)	**<0**.**001**	1.0 (0.9–1.0)	0.06
Lobe	1.1 (0.8–1.4)	0.54	0.9 (0.7–1.3)	0.91
Tumor diameter at CT	1.0 (1.0–1.1)	**0**.**01**	1.0 (0.9–1.1)	0.07
SUVmax tumor	0.3 (0.1–0.9)	**0**.**02**	0.5 (0.2–1.7)	0.26
Lymph node diameter at CT	1.4 (0.7–2.9)	0.40	1.1 (0.5–2.9)	0.79
SUVmax mediastinal lymph nodes	0.8 (0.4–1.7)	0.52	0.6 (0.2–1.6)	0.31
R classification	4.6 (2.2–9.6)	**<0** **.** **001**	4.5 (1.8–10.9)	**<0** **.** **001**

CI, confidence interval; CT, computed tomography; DFS, disease-free survival; HR, hazard ratio; OS, overall survival; VATS, video-assisted thoracic surgery; SUV, standardize uptake value.

Bold indicate statistical significative value.

**Table 6:** The multivariate analysis for DFS and OS in the N2 population.

Multivariate analysis	DFS		OS	
	HR (95% CI)	*p*-value	HR (95% CI)	*p*-value
Age	1.0 (0.9–1.1)	0.75	1.1 (0.9–1.1)	**0.05**
pT	1.5 (0.9–2.5)	0.13	2.0 (1.2–3.5)	**0.008**
pN2 subgroups	1.4 (0.9–2.4)	0.16	2.1 (1.2–3.7)	**0.007**
Lymph node ratio	1.0 (1.0–1.0)	**0.03**	—	—
Tumour diameter at CT	1.0 (0.9–1.1)	0.19	—	—
SUVmax tumor	0.4 (0.1–1.0)	0.06	—	—
R classification	3.2 (1.4–7.4)	**0.008**	2.0 (0.7–6.1)	**0.002**

CI, confidence interval; CT, computed tomography; DFS, disease-free survival; HR, hazard ratio; OS, overall survival; SUV, standardize uptake value.

Bold indicate statistical significative value.

The univariate analysis was also carried out in the R-u group (Table 7). We consider lymph node macro-metastases when the metastatic part is bigger than 2 mm. Variables that showed a tendency to be significant prognostic factors were pT [OS: HR 1.9 (95% CI 1.1–3.6), *p*-value = 0.02], pN2 subgroups [DFS: HR 1.7 (95% CI 0.9–2.9), *p*-value = 0.04; OS: HR 1.9 (95% CI 1.0–3.9), *p*-value = 0.03], number of resected lymph nodes [DFS: HR 0.9 (95% CI 0.9–1.0), *p*-value = 0.03], lymph node ratio [DFS: HR 1.0 (95% CI 1.0–1.0), *p*-value = 0.04], number of the positive lymph node in the higher station [DFS: HR 0.4 (95% CI 0.2–1.1), *p*-value = 0.03], macro-metastases [DFS: HR 30.5 (95% CI 3.9–240.0), *p*-value < 0.001; OS: HR 3.9 (95% CI 1.1–14.0), *p*-value = 0.01], and tumor diameter at CT scan [DFS: HR 1.0 (95% CI 1.0–1.1), *p*-value = 0.02]. In multivariable analysis, evaluated using the variables that had a significant *p*-value at univariate analyses, variables that confirmed to be significant prognostic factors were pT [OS: HR 1.9 (95% CI 0.9–3.7), *p*-value = 0.04] and macro-metastases (DFS: HR 28.8 (95% CI 3.5–239.5), *p*-value = 0.002; OS: HR 3.5 (95% CI 0.9–12.6), *p*-value = 0.05] (Table 8).

**Table 7:** The univariate analysis for DFS and OS in the R-u group.

Univariate analysis	DFS		OS	
	HR (95% CI)	*p*-value	HR (95% CI)	*p*-value
Age	1.0 (0.9–1.1)	0.97	0.6 (0.2–1.8)	0.39
Sex	0.9 (0.3–2.6)	0.9	1.0 (0.9–1.1)	0.31
Smoke	0.9 (0.3–2.9)	0.96	1.4 (0.4–4.9)	0.6
pT	1.5 (0.9–2.6)	0.1	1.9 (1.1–3.6)	**0.02**
pN2 subgroups	1.7 (0.9–2.9)	**0.04**	1.9 (1.0–3.9)	**0.03**
Histology	0.8 (0.2–2.7)	0.72	1.8 (0.5–6.3)	0.35
Open vs. VATS	1.3 (0.5–3.1)	0.57	1.1 (0.4–2.9)	0.9
No. lymph nodes	0.9 (0.9–1.0)	**0.03**	0.9 (0.9–1.0)	0.61
Lymph node ratio	1.0 (1.0–1.0)	**0.04**	1.0 (0.9–1.0)	0.57
No. lymph nodes higher station	1.1 (0.8–1.4)	0.64	1.2 (0.9–1.6)	0.12
Lymph node ratio higher station	0.9 (0.9–1.0)	0.15	1.6 (1.0–2.5)	0.35
No. positive lymph node higher station	0.4 (0.2–1.1)	**0.03**	1.2 (0.4–3.1)	0.78
Macro-metastases	30.5 (3.9–240.0)	**<0**.**001**	3.9 (1.1–14.0)	**0.01**
Lobe	1.1 (0.8–1.4)	0.53	1.1 (0.8–1.5)	0.56
Tumour diameter at CT	1.0 (1.0–1.1)	**0.02**	3.1 (0.7–13.7)	0.08
SUVmax tumor	0.5 (0.2–1.7)	0.28	0.7 (0.2–2.5)	0.6
Lymph node diameter at CT	1.2 (0.5–2.9)	0.64	0.8 (0.3–2.3)	0.68
SUVmax mediastinal lymph nodes	0.7 (0.3–1.8)	0.49	0.5 (0.2–1.6)	0.21

CI, confidence interval; CT, computed tomography; DFS, disease-free survival; HR: hazard ratio; OS, overall survival; VATS, video-assisted thoracic surgery; SUV, standardize uptake value.

Bold indicate statistical significative value.

**Table 8:** The multivariate analysis for DFS and OS in the R-u group.

Multivariate analysis	DFS		OS	
	HR (95% CI)	*p*-value	HR (95% CI)	*p*-value
pT		—	1.9 (0.9–3.7)	**0.04**
pN2 subgroups	1.2 (0.6–2.6)	0.66	1.8 (0.8–3.7)	0.14
No. lymph nodes	0.9 (0.9–1.0)	0.09	—	—
Lymph node ratio	1.0 (0.9–1.0)	0.69	—	—
No. positive lymph node higher station	0.5 (0.2–1.4)	0.16	—	—
Macro-metastases	28.8 (3.5–239.5)	**0.002**	3.5 (0.9–12.6)	**0.05**
Tumour diameter at CT	1.0 (0.9–1.1)	0.8	—	—

CI, confidence interval; CT, computed tomography; DFS, disease-free survival; HR, hazard ratio; OS, overall survival.

Bold indicate statistical significative value.

## Discussion

It is known that the diffusion of metastatic cells through the lymphatic pathway generally follows a specific pattern: intrapulmonary nodes, hilar nodes (N1), mediastinal nodes (N2) in the caudal–cranial direction, final to the supraclavicular (N3) nodes and distant organs (12). As previous studies evaluated, the pN2 groups are heterogeneous with regard to prognosis. The difference could come from the number of involved lymph nodes and stations and the specific patterns of lymphatic spread (13, 14).

At the time of surgery, the highest mediastinal lymph node resected represents the margin of cancer dissemination. Therefore, a metastasis in this station could be considered as a positive margin. The rationale lies in the possibility of cranial lymph node involvement or distant micro-metastases. Thereby, the involvement of cervical nodes or a more extensive mediastinal involvement may be underestimated. The finding of metastasis in the highest mediastinal lymph node dissected might be an important parameter to differentiate this subgroup of patients with a poor prognosis.

Previous studies investigated the difference in prognosis between complete and uncertain resection. In the study by Zheng et al., R0 and R-u 5-year survival rate and median survival time were 29% and 36.48 months vs. 13% and 24.43 months, respectively (*p* < 0.0001) (6). Osarogiagbon et al. found similar results: mean OS in R0 was 62 months, while in R-u it was 32 months (*p*-value < 0.0001) (15). In the literature, few studies paid attention to the prognostic impact of the highest mediastinal lymph node metastases and, as previously said, the definition of HMLN varied among studies. Gagliasso et al. used the IASLC definition of HMLN, so they found a better 5-year survival rate compared to less rigorous lymph node evaluation and carcinoma *in situ* in the bronchial margin (28.8% vs. 44.2% and 40.0%, respectively) (16).

Ren et al., following the IASLC definition, considered being HMLN positive as an independent risk factor for DFS and OS: among patients with N2 metastases, those with positive HMLN had significantly worse survival compared to R0 (DFS: 36 vs. 44 months, *p* < 0.001; OS: 50 vs. 59 months, *p* < 0.001) (17).

Sakao et al. defined HMLN as nodes lying above a horizontal line at the upper rim of the left innominate vein. They found that, in patients with advanced N2 disease, patients with positive HMLN had a 5-year survival rate of 21.0% compared to 52.0% of negative HMLN. Furthermore, patients with negative HMLN, even if they have multilevel N2 status, positive cN status, or T2–3 tumor status, had a better prognosis (18).

Two studies found no prognostic difference regarding survival between complete and incomplete resection. Both studies used a stricter definition of HMLN: for the right side they considered 2R, and for the left side 4l, 5, or 6. In these studies, the metastasis on HMLN did not show survival differences in completely resected N2 NSCLC (19, 20).

The results of our study support the idea that the presence of metastases in the highest mediastinal lymph node among those resected is a negative prognostic factor for DFS and OS. R-u had a higher recurrence and mortality rate compared to R0.

This study has some limitations. First, the retrospective nature of the study and the small cohort of patients may have affected the validity of the study. The category of R-u is wide; we focused only on the metastases on the highest mediastinal lymph nodes. Other studies should be done to compare the prognostic value of these subgroups and to evaluate the necessity of a specific adjuvant therapy. Future prospective for developing a preoperative evaluation of the lymphatic pathway and the sentinel node might be useful (21). So far, we suggest a systematic lymph node dissection for a better staging of the mediastinal lymph node status.

## Conclusion

The group of pN2 is not homogeneous from a prognostic point of view. R classification proposed by IASLC showed a significant improvement in survival discrimination. R-u delineates the crossing area between complete resection (R0) and incomplete resection (R1 and R2). The R-u for the presence of metastasis in the HMLN removed seems to be an independent prognostic factor for mortality and recurrence. The definition of HMLN varied among studies, whereby standardization of the definition was needed. The finding of metastasis in the HMLN represents the margin of cancer dissemination at the time of surgery, so it could imply metastasis into the N3 node or distant metastasis.

## Data Availability

The raw data supporting the conclusions of this article will be made available by the authors, without undue reservation.
